# ITS1 Copy Number Varies among *Batrachochytrium dendrobatidis* Strains: Implications for qPCR Estimates of Infection Intensity from Field-Collected Amphibian Skin Swabs

**DOI:** 10.1371/journal.pone.0059499

**Published:** 2013-03-21

**Authors:** Ana V. Longo, David Rodriguez, Domingos da Silva Leite, Luís Felipe Toledo, Cinthya Mendoza Almeralla, Patricia A. Burrowes, Kelly R. Zamudio

**Affiliations:** 1 Department of Ecology and Evolutionary Biology, Cornell University, Ithaca, New York, United States of America; 2 Laboratório de Antígenos Bacterianos II, Departamento de Genética, Evolução e Bioagentes, Instituto de Biologia, Unicamp, Campinas, Brazil; 3 Museu de Zoologia “prof. Adão José Cardoso”, Instituto de Biologia, Unicamp, Campinas, Brazil; 4 Departamento de Zoología, Instituto de Biología, Universidad Nacional Autónoma de México, México DF, México; 5 Department of Biology, University of Puerto Rico, San Juan, Puerto Rico; Ghent University, Belgium

## Abstract

Genomic studies of the amphibian-killing fungus (*Batrachochytrium dendrobatidis*, [*Bd*]) identified three highly divergent genetic lineages, only one of which has a global distribution. *Bd* strains within these linages show variable genomic content due to differential loss of heterozygosity and recombination. The current quantitative polymerase chain reaction (qPCR) protocol to detect the fungus from amphibian skin swabs targets the intergenic transcribed spacer 1 (ITS1) region using a TaqMan fluorescent probe specific to *Bd*. We investigated the consequences of genomic differences in the quantification of ITS1 from eight distinct *Bd* strains, including representatives from North America, South America, the Caribbean, and Australia. To test for potential differences in amplification, we compared qPCR standards made from *Bd* zoospore counts for each strain, and showed that they differ significantly in amplification rates. To test potential mechanisms leading to strain differences in qPCR reaction parameters (slope and y-intercept), we: a) compared standard curves from the same strains made from extracted *Bd* genomic DNA in equimolar solutions, b) quantified the number of ITS1 copies per zoospore using a standard curve made from PCR-amplicons of the ITS1 region, and c) cloned and sequenced PCR-amplified ITS1 regions from these same strains to verify the presence of the probe site in all haplotypes. We found high strain variability in ITS1 copy number, ranging from 10 to 144 copies per single zoospore. Our results indicate that genome size might explain strain differences in ITS1 copy number, but not ITS1 sequence variation because the probe-binding site and primers were conserved across all haplotypes. For standards constructed from uncharacterized *Bd* strains, we recommend the use of single ITS1 PCR-amplicons as the absolute standard in conjunction with current quantitative assays to inform on copy number variation and provide universal estimates of pathogen zoospore loads from field-caught amphibians.

## Introduction

Advances in quantitative polymerase chain reaction (qPCR) protocols and their application in detection and quantification of pathogens have contributed significantly to our understanding of disease dynamics in natural host populations [1], [2]. Disease ecologists investigating the amphibian-killing fungus (*Batrachochytrium dendrobatidis* [*Bd*]) use qPCR to detect the pathogen on the skin of wild amphibian populations, providing a non-invasive sampling method that can yield diagnosis within a few hours [2], [3]. *Bd* detection via qPCR has allowed researchers to detect infection levels in natural populations at different stages of emerging epidemics [4], track outbreaks that cause amphibian declines [5], establish disease thresholds predicting frog mortality [6], and reconstruct historical *Bd* epizootic waves spreading through naïve populations [7].

Recent genomic characterization of 20 global *Bd* strains indicates that *Bd* is composed of at least three divergent genetic lineages that differ in virulence [8]. One of these lineages, the global panzootic lineage (GPL) is hypervirulent and has been implicated in the recent epizootics [8]. In addition, a novel *Bd* strain recently discovered in Brazil differs in DNA content compared to GPL strains from Panama and California [9]. If these deeply-divergent strains carry polymorphisms at the primer or probe binding sites or if target ITS1 genes vary in copy number, then qPCR efficiency and sensitivity among strains may also vary [1], [10], which will reduce the comparability of qPCR infection intensity estimates across sites.

To generate standards for quantification of *Bd* via qPCR, researchers count zoospores from cultured *Bd* strains, extract genomic DNA (gDNA), and serially dilute to the desired concentrations (usually 100 to 0.1 zoospore genomic equivalents [2], [3]). The forward primer/probe combination of the qPCR TaqMan assay anneals to the internal transcribed spacer (ITS1) region, which is a rapidly evolving nuclear ribosomal repeat unit used for species-level identification [3], [11]. In fungal genomes, this region occurs in multiple copies providing over 100 potential primer/probe binding sites per haploid genome [3] and in *Bd* it can be repeated up to 169 times [12]. Duplications or deletions of genomic regions that include ITS1 sequences may result in over- or underestimation of zoospore load by established qPCR methods [3] because fluorescence and copy number in template DNA are linearly related.

In this study, we quantified and characterized ITS1 regions in multiple *Bd* strains to evaluate the effects of copy number and sequence variation on qPCR efficiency and zoospore quantification among strains. We quantified three different template preparations for each *Bd* strain including 1) genomic DNA (zoospore counts), 2) equimolar DNA solutions, and 3) ITS1 PCR amplicons. For each strain template, we tested differences in cycle threshold (C_t_), defined as the point on the amplification curve associated with exponential growth of PCR product. We then used ITS1 PCR amplicons as a standard to quantify the ITS1 copy number from our focal strains. Finally, we cloned and Sanger-sequenced ITS1 PCR amplicons to compare ITS1 haplotype diversity among strains, which could lead to differences in amplification rates. Our study highlights the importance of understanding the evolutionary history of *Bd* at each sampling locality and the caveats of using genomic DNA as a standard for qPCR. We include a step-by-step protocol (Supporting Information) so *Bd* researchers can measure ITS1 copy numbers from any uncharacterized *Bd* strain and estimate infection intensity from field-collected amphibian skin swabs.

## Methods

### *Bd* Strain Selection

We used *Bd* strains isolated from amphibian hosts in different countries to generate replicate sets of DNA standards for qPCR: JEL404 (USA, Maine), MexMkt (Mexico), JEL427 (Puerto Rico, Luquillo), PAB01 (Puerto Rico, Maricao), LFT001_01 (Brazil, São Paulo), CLFT023 (Brazil, Minas Gerais), CLFT024 (Brazil, Paraná), and LBabercrom (Australia). Most of these *Bd* strains belong to the global panzootic lineage (GPL, sensu Farrer et al. [8]) except LFT001_01 which is a highly divergent strain of *Bd* [9]. Therefore, our strain selection encompasses much of the known global genetic variation in *Bd.* In addition, our focal strains include *Bd* isolated from the same host species at small spatial scales (*e.g.* Puerto Rican *Eleutherodactylus coqui*, JEL427 and PAB01) to others cultured from different host species across the New World (JEL404, MexMkt and Brazilian strains: LFT001, CLFT023, and CLFT024). Because *Bd* quantification methods were developed using Australian strains [2], [3], [13], we included LBabercrom for comparison.

### DNA Extraction from *Bd* Cultures

We grew *Bd* strains on 1% mTGh agar with penicillin-G and streptomycin sulfate [14]. We flooded five-day-old colonies from at least five separate plates with distilled water [14], and pooled all harvested zoospores for use in standards. The zoospore solution was filtered using a 10 µm filter (Pall Acrodisc syringe filter AP-4001T) to ensure that zoosporangia were not present in the suspension. Using a 5% iodine solution (one part 10% povidone iodine: one part distilled water) we killed and stained a small aliquot (50 µL) of the zoospores to facilitate counting in a hemocytometer. We performed 10 individual counts for each strain and used the average count value to estimate the concentration of zoospores. We used these concentrations to make five to ten aliquots of 10^7^ zoospores mL^−1^ for each strain. We then centrifuged all the aliquots at high speed (13,000×g) for five minutes to obtain a zoospore pellet. We removed the supernatant and extracted the DNA from the pellet using 200 µL Prepman Ultra (Applied Biosystems, Inc.) following Boyle et al. [3], and measured the concentration of DNA in three extracts per strain using Qubit® double strand DNA high sensitivity assay (Life Technologies, Inc) (Table 1).

**Table 1:** Standard curve equations, reaction efficiencies and starting DNA concentrations of eight *Batrachochytrium dendrobatidis* strains.

Strain	Origin	DNAConcentration(ng/µL) ± SD	Standard Set A:Zoospore Counts (R^2^)	Efficiency(10^(−1/slope)^–1×100%)	Standard Set B: DNAdilutions (R^2^)	Efficiency (%)
**JEL404** *	USA (Maine)	0.33±0.01	*y* = −3.13x +31.6 (0.990)	109%	*y* = −3.42x +14.3 (0.999)	96.1%
**MexMkt**	Mexico	1.01±0.04	*y* = −3.31x +33.9 (0.999)	101%	*y* = −3.48x +15.9 (0.996)	93.8%
**JEL427**	Puerto Rico	2.76±0.06	*y* = −3.15x +30.8 (0.998)	108%	*y* = −3.44x +14.5 (0.999)	95.3%
**PAB01** *	Puerto Rico	0.65±0.04	*y* = −3.05x +30.8 (0.992)	113%	*y* = −3.28x +16.3 (0.997)	102%
**LFT001_01**	Brazil	4.11±0.10	*y* = −3.36x +30.5 (0.999)	98.4%	*y* = −3.48x +14.3 (0.999)	93.8%
**CLFT023**	Brazil	2.09±0.05	*y* = −3.35x +30.4 (0.993)	98.8%	*y* = −3.36x +13.2 (0.999)	98.4%
**CLFT024** *	Brazil	4.64±0.03	*y* = −3.35x +30.5 (0.993)	98.8%	*y* = −3.52x +15.1 (0.999)	92.3%
**LBAbercrom**	Australia	1.34±0.05	*y* = −3.22x +30.4 (0.997)	104%	*y* = −3.55x +12.6 (0.999)	91.3%

Standard curve coefficients (slopes and intercepts) were estimated from the linear regression equation between cycle threshold (Ct) and log-transformed zoospore counts. Average (± SD) DNA concentration of extracts is based on 10^7^ zoospores in 200 µL extraction buffer.

* 10^6^ zoospores.

### *Bd* Strain Comparison, ITS1 Copy Number and Real-time qPCR TaqMan Assay

**Figure 1:**
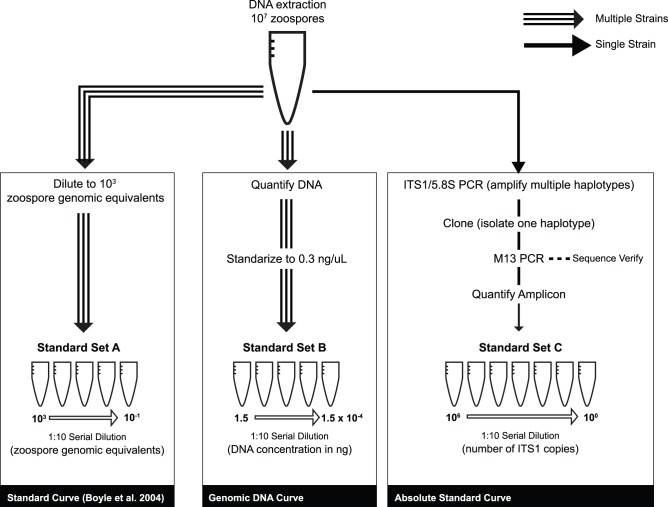
Schematic view of standard set preparations used in this study.

Our goal was to evaluate the qPCR assay performance parameters (slope and intercept) for several *Bd* strain preparations to compare their efficiency as absolute quantification standards (Fig. 1). qPCR performance parameters are important in determining whether the target PCR product successfully doubles with each amplification cycle [1], [15]. To test for potential biases during the DNA extraction and PCR amplification, we compared two standard sets made from PrepMan extracts: the first quantified by direct zoospore counts (Standard Set A) and the second based on equimolar DNA concentration dilutions for each strain (Standard Set B). If these two standard sets vary in amplification rates or in performance parameters among different *Bd* strains, we can infer that either the zoospore extraction efficiencies differ between the strains, or that genomic changes among strains cause differences in their amplification efficiencies.

For Standard Set A (based on zoospore counts), we assumed 100% extraction efficiency and serially diluted to the required concentrations following Boyle et al. [3]. We quantified DNA extracts of 10^7^ zoospores mL^−1^ three times using a Qubit®. For Standard Set A, we used a 5-log dynamic range starting at 10^3^ zoospore genomic equivalents in 5 µL (Fig. 1). For Standard Set B (based on equimolar DNA concentrations) we diluted the DNA extracts of each strain to make an aliquot with a final concentration of 0.3 ng µL^−1^, which is within the detectable range of the Qubit®. This amount of DNA roughly contains the genetic material of ∼6,000 zoospores µL^−1^ (based on a 46 Mb diploid genome size [16]). For Standard Set B we used a 5-log dynamic range starting at 0.3 ng µL^−1^ (Fig. 1).

We performed endpoint PCR on strain JEL427 (Puerto Rico) using primers ITS1-3 Chytr and 5.8S Chytr (hereafter ITS1/5.8S region, ∼146 bp) [3]. The resulting amplicons were cloned into the pGEM-T Easy Vector (Promega) and transformed into *E. coli* competent cells. To verify that bacterial cells incorporated the insert, we used blue-white screening, amplified the plasmid using M13 primers, and visualized the products on a 1.75% agarose gel. We cleaned the PCR products with ExoSap and quantified them with the Qubit® to estimate the mass of a fragment containing one copy of the ITS1/5.8S region. We estimated that this fragment weighs ∼4.10×10^−10^ ng, based on the total length of the M13 PCR-amplicon fragment (400 bp, including the 146 bp insert plus 254 bp of flanking vector sequence) and the average weight of one base pair (660 Da). Then, we built our third serial dilution (Standard Set C: PCR Amplicon, Fig. 1) with a 5-log dynamic range starting at 2.0×10^5^ copies of ITS1 µL^−1^. We used Standard Set C in qPCR reactions to infer the absolute number of ITS1 copies found in the 10^3^ zoospore extracts for each strain (based on zoospore counts in Standard Set A) and in 1.5 ng of DNA (Standard Set B).

We modified the qPCR protocol in Boyle et al. (2004) by adding BSA [13] and using Fast Advanced Master Mix (Applied Biosystems Inc.). We verified qPCR runs by performing duplicate reactions for each standard on an Applied Biosystems ViiA7. We did not set a standard curve *a priori*, rather we included Standard Set C in all qPCR runs and measured the cycle threshold (C_t_) value for each of the dilution curves in Standard Sets A and B. We considered two threshold values in our analyses, the automatic threshold set by the ViiA7 software and a custom threshold of 0.1 to standardize among different runs.

### ITS1 Haplotype Diversity

To assess ITS haplotype diversity among strains, we cleaned 400-bp PCR amplicons (M13+ ITS1/5.8S region) using ExoSAP and sequenced 21–43 clones per strain using Big Dye v3.1 chemistry on an ABI 3730 sequencer (Applied Biosystems, Inc.). We aligned and edited chromatograms using Sequencher (Gene Codes Corp.), identified all unique haplotypes, and calculated haplotype frequency [defined as the number of sequences of particular haplotype over the total number of sequences for each strain]. We also verified the presence of the probe-binding site in each haplotype sequence.

We searched whole genome sequence data (Rosenblum et al. *unpublished*) for strains MexMkt (Mexico), JEL427 (Puerto Rico), LFT001_01 (Brazil), CLFT023 (Brazil), CLFT024 (Brazil), and LBAbercrom (Australia) to assess whether the ITS1 haplotypes detected through cloning occurred in similar frequencies as in the genomic data. To estimate ITS1 haplotype frequencies in *Bd* genomes, we counted the number of times that each haplotype sequence recovered through cloning (truncated to a variable 35 bp fragment, see Fig. S1) was found among raw Illumina reads for each strain.

### Statistical Analyses

Using analyses of covariance, we tested for the effect of strains in the relationship between the number of genomic equivalents and Ct by fitting separate linear regressions for each standard set. If amplification efficiency among strains varied, we expected significantly different slopes and significant interaction terms across strains. If the number of ITS1 regions varied among strains, we expected differences in the y-intercept of the regression lines. We applied Tukey’s Honestly Significant Differences (HSD) to determine which pairwise strain comparisons were statistically different. To quantify the number of ITS1/5.8S regions in each strain, we used a standard curve based on the ITS1 PCR-amplicon (Standard Set C) against each of the zoospore-based serial dilutions (Standard Set A and Standard Set B). We performed all statistical analyses using R [17].

We compared the number of ITS1 haplotypes recovered by cloning and whole genome sequencing using a paired t-test. Finally, we calculated ITS1 haplotype frequencies for both data sets and estimated pairwise genetic differentiation among the ITS1 haplotypes for each *Bd* strain using *F_ST_* statistics implemented in Arlequin 3.5 [18].

## Results

### Differences in C_t_ Values from Zoospore Counts (Standard Set A) and Equimolar DNA Dilutions (Standard Set B)

**Figure 2:**
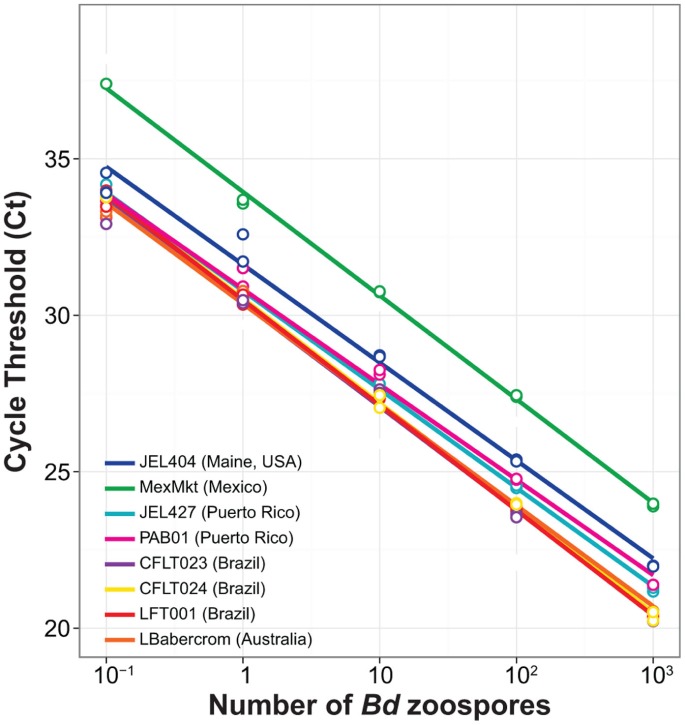
Standard curve regression lines for multiple isolates of *Batrachochytrium dendrobatidis* based on zoospore counts (Standard Set A).

As expected, we found a significantly negative relationship between the number of zoospores and cycle threshold number, C_t_ (*Β* = −3.31, *T* = −44.93, *P*<0.000, Fig. 2). The best-fit regression model showed independent slopes and intercepts among *Bd* strains, and a significant interaction term between log10-transformed zoospore number and strain (*F*_7 = _2.64, *P* = 0.018) (Table 1). All standard curves made from zoospore counts (Standard Set A) exhibited similar intercepts, except the MexMkt (Mexico) strain (*F*_7 = _134.5, *P*<0.001, Fig. 2). MexMkt showed a higher intercept value for any given zoospore quantity indicating that this strain has fewer ITS1 copies per zoospore. The interaction effect between slope and intercept was driven by a significantly lower slope in strain PAB01 (Puerto Rico) (*Β* = −3.05, *T* = 2.55, *P* = 0.013) and a marginally lower slope in JEL404 (USA) (*Β* = −3.13, *T* = 1.76, *P* = 0.083). Therefore, most strains had similar amplification efficiencies (>98%) with the exception of PAB01 (Puerto Rico) and JEL404 (USA) which showed slightly higher efficiencies (Table 1).

The same starting number of zoospores (10^7^) extracted from each *Bd* strain had different DNA concentrations, that ranged from 1.01 to 4.11 ng µL^−1^ based on Qubit® quantification (Table 1). These differences among strains could occur by four possible mechanisms: (1) contamination with other microorganisms, (2) error or low resolution in DNA quantification, (3) differences in DNA extraction efficiency, or (4) different genome sizes. We grew all cultures on antibiotics and did not observe other microorganisms while counting zoospores; therefore, it is unlikely that contamination alone accounts for the large differences across strains. We ruled out the second hypothesis, because we found only negligible variation among three replicate Qubit® measurements for each of the three 10^7^ zoospore extracts per strain (Table 1). Finally, the third scenario is also unlikely, because we simultaneously extracted all strains using the same extraction reagents and protocols. These differences in amplification rates remained even after diluting all strains to the same DNA concentration (Standard Set B); thus, we can infer that differences in *Bd* genome size underlie the biases in quantification of DNA template among strains.

**Figure 3:**
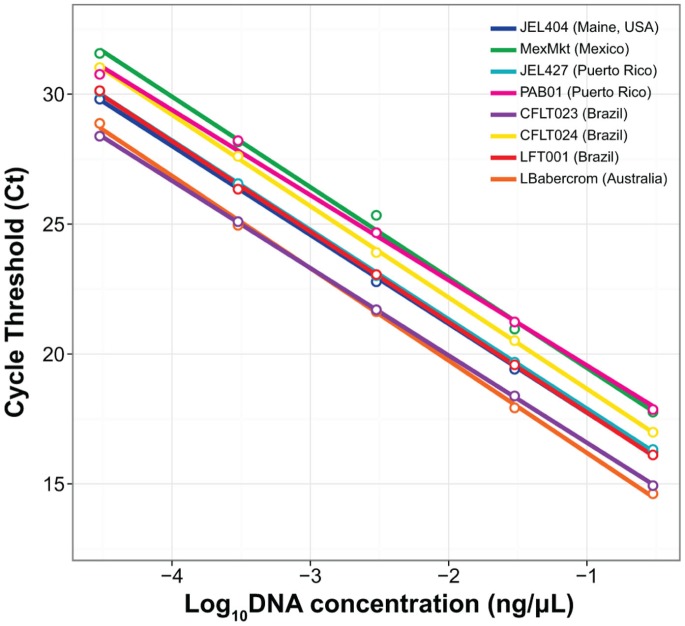
Standard curve regression lines for multiple strains of *Batrachochytrium dendrobatidis* based on equimolar DNA solutions starting at 0.3 ng µL^−1^ (Standard Set B).

qPCR of equimolar *Bd* DNA dilutions (Standard Set B) showed a significant negative relationship between nanograms of DNA and Ct (*Β* = −3.34, *T* = −135.99, *P*<0.001, Fig. 3). In contrast to the zoospore regression models, the best model fitted independent intercepts for every strain in our study (*F*_7 = _161.6, *P*<0.001, Fig. 3). All standard curves made from equimolar DNA concentrations (total of 1.5 ng) showed significant departures in their intercepts from strain LBabercrom (Australia), except the curve from Brazil CLFT023 (*Β* = 0.09, *T* = 0.69, *P* = 0.50). Our post-hoc Tukey's HSD tests revealed further pairwise similarities between strains in amplification profiles: JEL404-JEL427, LFT001_01-JEL427, LFT001_01-JEL404, and PAB01-MexMkt did not significantly differ from each other (Fig. 3). Strain MexMkt and LBabercrom showed the largest differences in intercept C_t_ values (Fig. 3).

### ITS1/5.8S Copy Number and Genome Size Varied among *Bd* Strains

**Figure 4:**
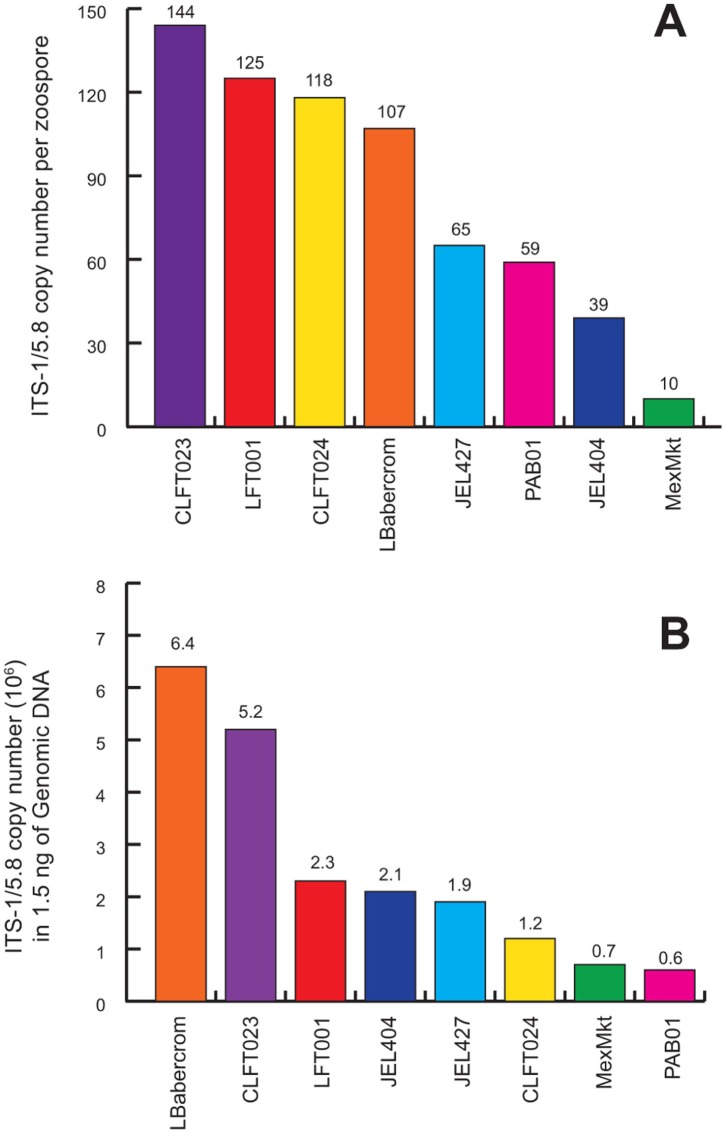
Estimated ITS1 copy number variation for each *Batrachochytrium dendrobatidis* isolate using PCR amplicons in (A) one zoospore, and (B) in 1.5 ng DNA.

We compared the differences in ITS1 copy number among *Bd* strains by using the absolute standard (Standard Set C) and holding zoospore quantity or input DNA constant (Fig. 4). When we held zoospore number constant (10^3^), our results showed high copy number variability, which ranged from 10 copies in MexMkt to 144 copies in CLFT023 (Fig. 4A). When we held the DNA concentration constant (1.5 ng), strains also showed 10-fold differences in copy number ranging from 6.0×10^5^ to 6.0×10^6^ (Fig. 4B). Based on these two measures of copy number, the inferred DNA content per zoospore was significantly different among strains (Fig. S2).

### Differences in ITS1 Haplotype Diversity

**Figure 5:**
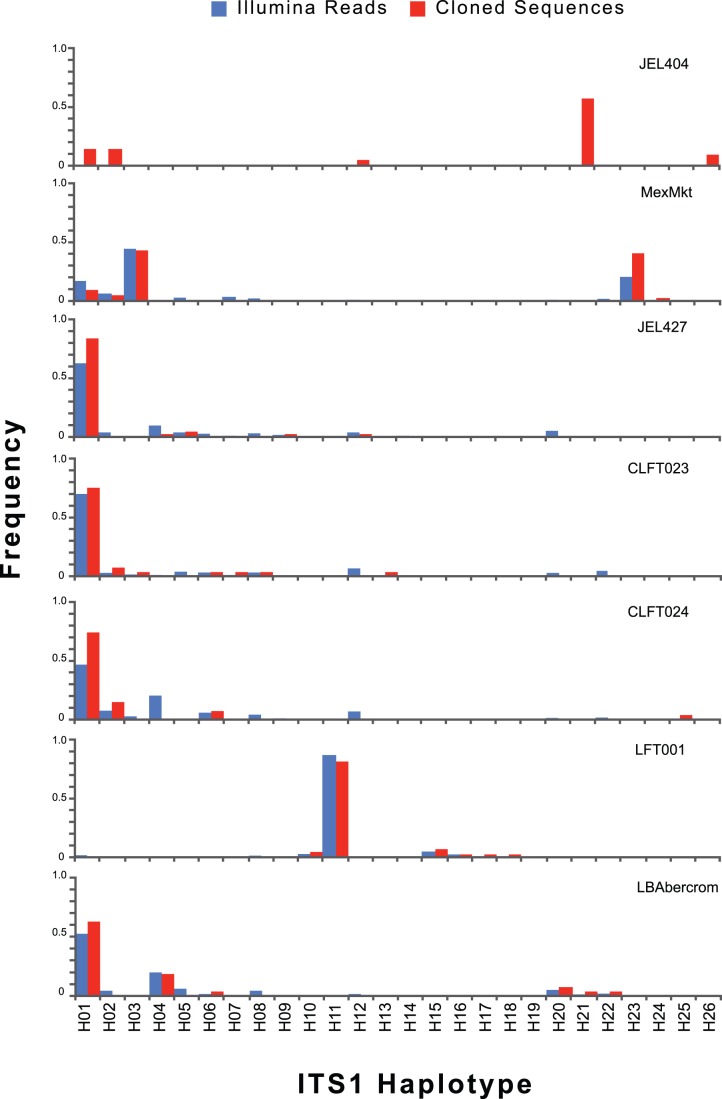
ITS1 haplotype frequencies for each strain. Proportions were estimated using Illumina sequencing (blue) and cloning/Sanger sequencing (red).

**Figure 6:**
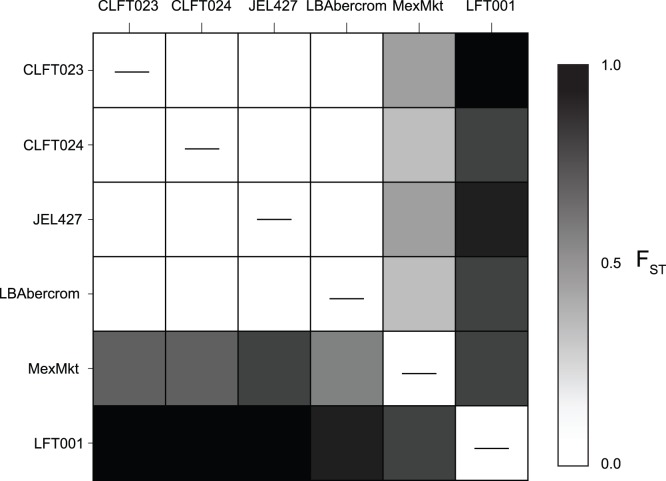
Pairwise *F*_ST_ values based on ITS1 haplotype frequencies in each *Bd* strain. Upper and lower diagonals are pairwise *F*_ST_ values estimated from ITS1 haplotypes obtained by cloning/Sanger sequencing, and from Illumina sequencing, respectively. Darker shades represent greater genetic differentiation.

We detected 26 different ITS1 haplotypes among seven strains through cloning (Fig. S1, Appendix S1). Sequence variants were conserved from bases 1 to 42, which included the primers and the Chytr MGB2 probe binding site, and bases 126 to 154 (Fig. S1). The area internal to these conserved regions showed multiple indels and base changes, resulting in unique haplotypes that vary in length (136–152 bp, Fig. S1). We did not detect significant changes in haplotype frequency distributions obtained by cloning and by Illumina sequencing (Fig. 5 and Fig. 6). However, Illumina sequencing detected significantly more haplotypes (11.2±9.3) per strain on average compared to cloning (5.8±1.1) (*T* = 4.53, *DF* = 6.9, *P* = 0.003). This result indicates that cloning did not bias our relative estimates of haplotype diversity but failed to detect rare haplotypes. Strains MexMkt and LFT001_01 had different haplotype frequency distributions compared to JEL427, CLFT023, CLFT024, and LBabercrom (Fig. 5), which had similar frequency profiles (average *F*_ST_ = 0.01, Fig. 6, Table S2).

## Discussion

Our results show that using zoospores counts (Standard Set A) to establish qPCR standard curves can be problematic because *Bd* strains vary in their ITS1 copy number (Fig. 2). These strain differences in gene copy number directly affect qPCR estimates of pathogen load from amphibians or the environment, and thus, underscore the need to improve our current diagnostic methods. Differences in copy number persisted even after holding *Bd* DNA concentrations constant (Standard Set B, Fig. 3), likely due to genetic changes caused by genomic duplications or deletions. On the other hand, haplotype diversity does not appear to affect quantification, because none of the haplotypes detected by cloning had changes at the probe-binding site (Fig. S1). Our results indicate that researchers interested in estimating absolute *Bd* zoospore load from amphibians or the environment will have to incorporate an independent measure to estimate ITS1 copy number from the strain used as a standard.

Our comparison of *Bd* qPCR standards made from strains collected in six countries points to patterns of genomic change during the evolution and global spread of *Bd* [8]. In the strain MexMkt (Mexico) we found significantly higher cycle threshold values for any given number of zoospores even after holding DNA concentration constant (Fig. 3), suggesting a decrease in the number of probe/primer binding sites in the genome of that strain. Potential causes for a decrease in probe binding include: (1) inhibitors in the qPCR reaction that reduce affinity between probe and binding site, (2) changes in structural conformation or mutations that affect probe/primer binding sites, or (3) copy number/genome size variation. We can eliminate inhibition in our qPCR reaction, because the slope of MexMkt showed an efficiency value of 101% (Table 1). Differences in structural conformations are not expected to interfere with amplification because the ITS1-3/5.8S Chytr primers were designed to avoid G-rich stretches and AT-rich stem/loop structures [3]. Likewise, Illumina reads showed no polymorphism in ITS primer binding sites in MexMkt or any other *Bd* strain (Table S1), thus differential primer binding is not causing variation in amplification in our focal strains. The MexMkt strain showed only ten ITS1 copies in a single zoospore, compared to 39–144 copies detected in other strains (Fig. 4A). Thus, our data indicate that these differences are most likely due to a reduction in genome size (Fig. S2).

All other strains showed similar slopes and intercepts for zoospore-based standard curves (Standard Set A; Table 1). Differences in ITS1 copy number (39–144 copies) were not apparent using Standard Set A, but became evident as more (or less) zoospores were required to attain 1.5 ng of DNA (Standard Set B, Fig. 4B). For example, strain LBabercrom had 107 ITS1 copies in a single zoospore, but showed the highest number of copies (6.4×10^4^) in 1.5 ng of extracted DNA (Fig. 4). This finding indicates that LBabercrom may have a low DNA content per zoospore but includes many copies of the ITS1 region (Fig. S2). In contrast, strain CLFT024 has a large estimated genome with 118 ITS1 copies per zoospore; thus, the ratio of ITS1 to genome size may not be constant across *Bd* strains. Although intraspecific variability in *Bd* ITS1 regions often occurred among strains from different countries, our results also highlighted local differences in ITS1 copy number and haplotype diversity (Fig. 4A and Fig. 5). For instance, strains LFT001_01, CLFT023, and CLFT024 were isolated from three states in Brazil (São Paulo, Minas Gerais and Paraná, respectively). These strains show similar ITS1 copy numbers (118–144, Fig. 4A) but LFT001_01 has a unique haplotype distribution (Fig. 5 and Fig. 6). Recent comparisons using flow cytometry of *Bd* strains from California (JEL270), Panama (JEL423), and Brazil (JEL648, UM142) also indicated significant variation in DNA content [9], and the two fully sequenced *Bd* strains JEL423 and JAM81 have different assembly sizes, which may result from the presence of chromosomal length polymorphisms [16]. Thus, our results corroborate these earlier findings of genome size evolution within *Bd*, and indicate that insertions and/or deletions of regions including ribosomal ITS1 may explain the differences we observed in qPCR standard curves across strains.

ITS1 copy number variation among *Bd* lineages has important implications for comparative studies of disease dynamics in spatially isolated amphibian populations. Infection intensity thresholds of approximately 10,000 *Bd* genomic equivalents (ge; or zoospores) are often associated with disease epidemics, mortality, and population extirpations [5], [7], [19]. However, other species carrying a 10-fold lower average infection intensity also experienced severe die-offs [20]. Before assigning biological causation to these observed thresholds, researchers will need to evaluate bias in qPCR measurements resulting from different *Bd* standards.

10,000 zoospores and 1,000 zoospores can generate the same amount of fluorescence in qPCR assays if standards are made with *Bd* strains that have a 10-fold difference in the number of ITS1 regions. Furthermore, the possibility of sampling amphibians with co-infections of multiple strains with variable ITS1 copy number will remain a problem in wild populations, unless we characterize ITS1 regions and develop strain-specific assays. To reduce the potential biases caused by *Bd* genome variation or co-infections, we recommend that researchers combine PCR amplicon standards in conjunction with standards based on zoospore counts for detecting *Bd* [2], [3]. Researchers should report the ITS copy number per zoospore of the strain used as a qPCR standard, or ITS copy number per sample in addition to *Bd* genomic equivalents. PCR-amplicon standards can be generated cheaply and will allow researchers to determine the number of ITS1 regions in newly isolated *Bd* strains. Our strain-independent method (see Supporting Information) complements traditional standard curves, and provides accurate and comparable measures of infection intensities across sampling sites and studies.

## Supporting Information

Figure S1 An alignment of 26 ITS1 haplotypes detected by cloning and sequencing PCR products generated using primers ITS1-3 Chytr and 5.8S Chytr on genetic material from seven *Bd* strains. Light blue area represents unique 35 bp fragment used for searches in the Illumina sequence data. See for FASTA formatted sequences.
(EPS)

Figure S2 Estimated DNA content per zoospore from ITS copy number and the number of copies in 1.5 ng DNA.
(EPS)

Table S1 Percent identity and range in coverage for each strain at each primer binding site. NR represents the total number of filtered reads searched.
(DOCX)

Table S2 Pairwise *F_ST_* values. Upper diagonal values are from cloning/Sanger sequencing and lower diagonal values from Illumina sequencing. Significant *F_ST_* values (P<0.05) are shown in bold.
(DOCX)

Appendix S1 FASTA formatted ITS1 haplotypes with gaps included.
(DOCX)
