# Correction: ITS1 Copy Number Varies among *Batrachochytrium dendrobatidis* Strains: Implications for qPCR Estimates of Infection Intensity from Field-Collected Amphibian Skin Swabs

**DOI:** 10.1371/annotation/e4c527e6-4b6f-4c12-970c-a9e2e6ed8f79

**Published:** 2013-10-30

**Authors:** Ana V. Longo, David Rodriguez, Domingos da Silva Leite, Luís Felipe Toledo, Cinthya Mendoza Almeralla, Patricia A. Burrowes, Kelly R. Zamudio

The seventh author (KRZ) was not correctly indicated as the recipient of two grants from the National Science Foundation (DEB-0815315 and DEB-1120249). In addition, multiple sections were incorrectly omitted from the Supporting Information, and references used for this information were incorrectly omitted from the References List. A file containing the missing information and references can be found on the following page: http://www.plosone.org/corrections/pone.0059499.001.cn.docx

