# Supplementary material for: ITS1 Copy Number Varies among Batrachochytrium dendrobatidis Strains: Implications for qPCR Estimates of Infection Intensity from Field-Collected Amphibian Skin Swabs
Source: PLoS One. 2013 Mar 21;8(3):e59499. doi: 10.1371/journal.pone.0059499 (PMC3605245; doi:10.1371/journal.pone.0059499)
Supplement: Table S1 — Percent identity and range in coverage for each strain at each primer binding site. NR represents the total number of filtered reads searched. (DOCX) [file pone.0059499.s003.docx]

**Table S1.** Percent identity and range in coverage for each strain at each primer binding site. NR represents the total number of filtered reads searched.

| **Strain** | **ITS1-3 Chytr** | **5.8s Chytr** | **NR** |
| --- | --- | --- | --- |
| CLFT023 | 100 (22-148) | 100 (24-325) | 1.97E+07 |
| CLFT024 | 100 (161-803) | 99.98 (545-1625) | 4.70E+07 |
| JEL427 | 99.97 (30-411) | 100 (118-360) | 6.54E+06 |
| LBabercrom | 99.8 (36-299) | 99.98 (145-401) | 7.55E+06 |
| LFT001 | 99.9 (144-232) | 99.98 (583-971) | 1.12E+07 |
| MexMkt | 100 (34-386) | 100 (123-446) | 1.28E+07 |
