# Supplementary material for: ITS1 Copy Number Varies among Batrachochytrium dendrobatidis Strains: Implications for qPCR Estimates of Infection Intensity from Field-Collected Amphibian Skin Swabs
Source: PLoS One. 2013 Mar 21;8(3):e59499. doi: 10.1371/journal.pone.0059499 (PMC3605245; doi:10.1371/journal.pone.0059499)
Supplement: Table S2 — Pairwise FST values. Upper diagonal values are from cloning/Sanger sequencing and lower diagonal values from Illumina sequencing. Significant FST values (P<0.05) are shown in bold. (DOCX) [file pone.0059499.s004.docx]

**Table S2.** Pairwise *F_ST_* values. Upper diagonal values are from cloning/Sanger sequencing and lower diagonal values from Illumina sequencing. Significant *F_ST_* values (P < 0.05) are shown in bold.

|  | **CLFT023** | **CLFT024** | **JEL427** | **LBAbercrom** | **MexMkt** | **LFT001** |
| --- | --- | --- | --- | --- | --- | --- |
| **CLFT023** | - | 0.00 | **0.01** | **0.03** | **0.39** | **0.62** |
| **CLFT024** | 0.00 | - | **0.03** | **0.04** | **0.40** | **0.62** |
| **JEL427** | 0.01 | **0.03** | - | **0.07** | **0.49** | **0.68** |
| **LBAbercrom** | 0.03 | 0.04 | **0.07** | - | **0.34** | **0.56** |
| **MexMkt** | **0.39** | **0.40** | **0.49** | **0.34** | - | **0.50** |
| **LFT001** | **0.62** | **0.62** | **0.68** | **0.56** | **0.50** | - |
