# Supplementary material for: ITS1 Copy Number Varies among Batrachochytrium dendrobatidis Strains: Implications for qPCR Estimates of Infection Intensity from Field-Collected Amphibian Skin Swabs
Source: PLoS One. 2013 Mar 21;8(3):e59499. doi: 10.1371/journal.pone.0059499 (PMC3605245; doi:10.1371/journal.pone.0059499)
Supplement: Appendix S1 — FASTA formatted ITS1 haplotypes with gaps included. (DOCX) [file pone.0059499.s005.docx]

**Appendix SI. FASTA formatted ITS1 haplotypes with gaps included.**

>H11

CCTTGATATAATACAGTGTGCCATATGTCACGAGTCGAACAAAATTTATTTGTTTT-TTCGACAAATTTAATTGAAAT-GATT-----TAATTTAATTGAAAAATAATTGAAAA-AAATATTAAAAACAACTTTTGACAACGGATCTCTTGGCT

>H17

CCTTGATATAATACAGTGTGCCATATGTCACGAGTCGAACAAAATTTATTTGTTTT-TTCGACAAATTTAATTGAAAT-GATT-----TAATTTAATTGAAAAATAATTGAAAA-AAATATT-AAAACAACTTTTGACAACGGATCTCTTGGCT

>H18

CCTTGATATAATACAGTGTGCCATATGTCACGAGTCGAACAAAATTTATTTGTTTT-TTCGACAAATTTAATTGAAAT-GATT-----TAATTTAATTGAAAAATAATCGAAAA-AAATATTAAAAACAACTTTTGACAACGGATCTCTTGGCT

>H10

CCTTGATATAATACAGTGTGCCATATGTCACGAGTCGAACAAAATTTATTTGTTTT-TTCGACAAATTTAATTGAAAT-GATT-----TAATTTAATCCAAAA--AATATATTAAAAATATT-AAAACAACTTTTGACAACGGATCTCTTGGCT

>H15

CCTTGATATAATACAGTGTGCCATATGTCACGAGTCGAACAAAATTTATTTGTTTT-TTCGACAAATTTAATTGAAAT-GATT-----TAATTTAATCCAAAA--AAT--ATATTAAA----AAAAACAACTTTTGACAACGGATCTCTTGGCT

>H16

CCTTGATATAATACAGTGTGCCATATGTCACGAGTCGAACAAAATTTATTTGTTTT-TTCGACAAATTTAATTGAAAT-GATT----TTAATTTAATTGAAAAA-AATTGAAAA-AAATATTAAAAACAACTTTTGACAACGGATCTCTTGGCT

>H22

CCTTGATATAATACAGTGTGCCATATGTCACGAGTCGAACAAAATTTATTTATTTT-TTCGACAAATTAATTGGAAAT-GATT----TTAATTTAATTGAAAAA-AATTGAAAATAAATATTAAAAACAACTTTTGACAACGGATCTCTTGGCT

>H20

CCTTGATATAATACAGTGTGCCATATGTCACGAGTCGAACAAAATTTATTTATTTT-TTCGACAAATTAATTGGAAATTGAATGATTTTAATTTAATTGAAAA-TAATTGAAAATAAATATTAAAAACAACTTTTGACAACGGATCTCTTGGCT

>H08

CCTTGATATAATACAGTGTGCCATATGTCACGAGTCGAACAAAATTTATTTATTTT-TTCGACAAATTAATTGGAAATTGAATGATTTTAATTTAATTGAAAAA-AATTGAAAATAAATATTAAAAACAACTTTTGACAACGGATCTCTTGGCT

>H03

CCTTGATATAATACAGTGTGCCATATGTCACGAGTCGAACAAAATTTATTTATTTT-TTCGACAAATTAATTGGAAATTGAAT------AATTTAATTGAAAAATAATTGAAAATAAATATTAAAAACAACTTTTGACAACGGATCTCTTGGCT

>H26

CCTTGATATAATACAGTGTGCCATATGTCACGAGTCGAACAAAATTTATTTATTTT-TTCGACAAATTAATTGGAAATTGAAT------AATTTAATTGAAAA-TAATTGAAAATAAATATTAAAAACAACTTTTGACAACGGATCTCTTGGCT

>H12

CCTTGATATAATACAGTGTGCCATATGTCACGAGTCGAACAAAATTTATTTATTTT-TTCGACAAATTAATTGGAAATTGAATG------ATTTAATTGAAAAA-AATTGAAAATAAATATTAAAAACAACTTTTGACAACGGATCTCTTGGCT

>H24

CCTTGATATAATACAGTGTGCCATATGTCACGAGTCGAACAAAATTTATTTATTTT-TTCGACAAATTAATTGGAAGTTGAATG------ATTT-----------AATTGAAAATAAATATTAAAAACAACTTTTGACAACGGATCTCTTGGCT

>H23

CCTTGATATAATACAGTGTGCCATATGTCACGAGTCGAACAAAATTTATTTATTTT-TTCGACAAATTAATTGGAAATTGAATG------ATTT-----------AATTGAAAATAAATATTAAAAACAACTTTTGACAACGGATCTCTTGGCT

>H09

CCTTGATATAATACAGTGTGCCATATGTCACGAGTCGAACAAAATTTATTTATTTT-TTCGACAAATTAATTGGAAATTGAAT-------ATTTAATTGAAAAA-AATTGAAAATAAATATTAAAAACAACTTTTGACAACGGATCTCTTGGCT

>H01

CCTTGATATAATACAGTGTGCCATATGTCACGAGTCGAACAAAATTTATTTATTTT-TTCGACAAATTAATTGGAAATTGAAT------AATTTAATTGAAAAA-AATTGAAAATAAATATTAAAAACAACTTTTGACAACGGATCTCTTGGCT

>H04

CCTTGATATAATACAGTGTGCCATATGTCACGAGTCGAACAAAATTTATTTATTTT-TTCGACAAATTAATTGGAAATTGAATA-----ATTTTAATT------TAATTGAAAATAAATATTAAAAACAACTTTTGACAACGGATCTCTTGGCT

>H13

CCTTGATATAATACAGTGTGCCATATGTCACGAGTCGAACAAAATTTATTTATTTT-TTCGACAAATTAATTGGAAATTGAAT------AATTTAATTGAGAAA-AATTGAAAATAAATATTAAAAACAACTTTTGACAACGGATCTCTTGGCT

>H02

CCTTGATATAATACAGTGTGCCATATGTCACGAGTCGAACAAAATTTATTTATTTT-TTCGACAAATTAATTGGAAATTGAAT------AATTT-----------AATTGAAAATAAATATTAAAAACAACTTTTGACAACGGATCTCTTGGCT

>H19

CCTTGATATAATACAGTGTGCCATATGTCACGAGTCGAACAAAATTTATTTATTTT-TTCGACAAATTAATTGGAAATTGAATAGTTTTAATTT-----------AATTGAAAATAAATATTAAAAACAACTTTTGACAACGGATCTCTTGGCT

>H25

CCTTGATATAATACAGTGTGCCATATGTCACGAGTCGAACAAAATTTATTTATTTT-TTCGACAAATTAATTGGAAATTGAAT------AATTTAGTTGAAAAAAAATTGAAAATAAATATTAAAAACAACTTTTGACAACGGATCTCTTGGCT

>H06

CCTTGATATAATACAGTGTGCCATATGTCACGAGTCGAACAAAATTTATTTATTTT-TTCGACAAATTAATTGGAAATTTAAT------AATTTAATTGAAAAA-AATTGAAAATAAATATTAAAAACAACTTTTGACAACGGATCTCTTGGCT

>H05

CCTTGATATAATACAGTGTGCCATATGTCACGAGTCGAACAAAATTTATTTATTTT-TTCGACAAATTAATTGGAAATTGAAT------AATTTAATTGAAAAAAAATTGAAAATAAATATTAAAAACAACTTTTGACAACGGATCTCTTGGCT

>H14

CCTTGATATAATACAGTGTGCCATATGTCACGAGTCGAACAAAATTTATTTATTTTGTTCGACAAATTAATTGAAAATTGAAT------AATTTAATTGAAAAAAAATTGAAAATAAATATTAAAAACAACTTTTGACAACGGATCTCTTGGCT

>H07

CCTTGATATAATACAGTGTGCCATATGTCACGAGTCGAACAAAATTTATTTATTTT-TTCGACAAATTAATTGGAAATTGAAT------AATTTAATTGAAAAAAAATTGAAAATAAA-------AACAACTTTTGACAACGGATCTCTTGGCT

>H21

CCTTGATATAATACAGTGTGCCATATGTCACGAGTCGAACAAAATTTATTTATTTT-TTCGACAAATTAATTGGAAATTGAATAA-TTTAATTTAATTGAAAAA-AATTGAAAATAAATATTAAAAACAACTTTTGACAACGGATCTCTTGGCT
